# A New Tool to Study Parkinsonism in the Context of Aging: MPTP Intoxication in a Natural Model of Multimorbidity

**DOI:** 10.3390/ijms22094341

**Published:** 2021-04-21

**Authors:** Lorena Cuenca-Bermejo, Elisa Pizzichini, Valeria C. Gonçalves, María Guillén-Díaz, Elena Aguilar-Moñino, Consuelo Sánchez-Rodrigo, Ana-María González-Cuello, Emiliano Fernández-Villalba, María Trinidad Herrero

**Affiliations:** 1 Clinical and Experimental Neuroscience (NiCE), Institute for Aging Research (IUIE), School of Medicine, University of Murcia, 30100 Murcia, Spain; lorena.cuenca@um.es (L.C.-B.); pizzichini.1757373@studenti.uniroma1.it (E.P.); vaal.cassia@gmail.com (V.C.G.); maria.guillend@um.es (M.G.-D.); elena.aguilarm@um.es (E.A.-M.); consu.rodrigo@gmail.com (C.S.-R.); agcuello@um.es (A.-M.G.-C.); 2 Biomedical Research Institute of Murcia (IMIB-Arrixaca), Campus Mare Nostrum, University of Murcia, 30120 Murcia, Spain; 3 Department of Biology and Biotechnology “Charles Darwin” (BBCD), Sapienza, University of Rome, 00185 Rome, Italy; 4 Disciplina de Neurociência, Departamento de Neurologia e Neurocirurgia, Universidade Federal de São Paulo (UNIFESP), São Paulo 04039-032, Brazil

**Keywords:** *O. degus*, MPTP, Parkinson’s disease, aging, neurodegeneration, neuroinflammation, neurotoxicity

## Abstract

The diurnal rodent *Octodon degus* (*O. degus*) is considered an attractive natural model for Alzheimer’s disease and other human age-related features. However, it has not been explored so far if the *O. degus* could be used as a model to study Parkinson’s disease. To test this idea, 10 adult male *O. degus* were divided into control group and MPTP-intoxicated animals. Motor condition and cognition were examined. Dopaminergic degeneration was studied in the ventral mesencephalon and in the striatum. Neuroinflammation was also evaluated in the ventral mesencephalon, in the striatum and in the dorsal hippocampus. MPTP animals showed significant alterations in motor activity and in visuospatial memory. Postmortem analysis revealed a significant decrease in the number of dopaminergic neurons in the ventral mesencephalon of MPTP animals, although no differences were found in their striatal terminals. We observed a significant increase in neuroinflammatory responses in the mesencephalon, in the striatum and in the hippocampus of MPTP-intoxicated animals. Additionally, changes in the subcellular expression of the calcium-binding protein S100β were found in the astrocytes in the nigrostriatal pathway. These findings prove for the first time that *O. degus* are sensitive to MPTP intoxication and, therefore, is a suitable model for experimental Parkinsonism in the context of aging.

## 1. Introduction

In the last decades, Parkinson’s disease (PD) has become one of the neurodegenerative pathologies with the highest impact in our society [1]. The two principal histopathological hallmarks of this disorder are i) dopamine depletion (due to the death of dopaminergic neurons and the loss of their terminals in the striatum) and ii) proteinaceous inclusions (enriched in α-synuclein) in neuronal cytoplasm, known as Lewy bodies [2]. Other features underlying the pathogenesis of the disease include mitochondrial disruption, oxidative stress and the elevation of neuroinflammatory markers [3]. The main clinical manifestations of these brain cellular and molecular alterations are movement disorders (bradicinesia, rigidity, postural instability, resting tremor, constipation, among others) and non-motor symptoms, such as memory impairment, cognitive decline, olfactory or sleep dysfunctions [4,5]. 

Throughout the years, diverse approaches have been used to analyze different aspects of the disease, ranging from toxin-based models to transgenic animals. Currently, a model that fully recapitulates the human disease is not available yet, although all of the experimental approaches have advantages and limitations that must be considered when it comes to the study design [6]. Among the most commonly used models to mimic human PD, of great relevance is the experimental model based on the neurotoxin 1-methyl-4-phenyl-1,2,3,6-tetrahydro-pyridine (MPTP). Several lines of evidence have shown that MPTP reproduces motor and non-motor symptoms of PD, and it can simulate early and late disease stages of the disease depending on the administration regimen [6,7]. 

When MPTP is administered systemically, it acts as a pro-toxin that is able to cross the blood brain barrier. Then it is taken up by astrocytes, it is metabolized to MPP+, released and finally captured by the dopaminergic neurons of the ventral mesencephalon and their terminals in the striatum via dopamine transporters. Once inside the cell, MPP+ induces the disruption of the mitochondrial electron chain, which triggers oxidative stress and neurotoxic effects, with neuronal death as a result [8]. Additionally, several studies have shown that MPTP also induces an exacerbated inflammatory response, which has been shown to be a key element in neurodegeneration [9,10,11].

Administration regimens, as well as their characterization, have been well-established in monkeys and mice. Non-human primates are the most valuable experimental model due to their greater similarity to humans, but their use is limited by certain disadvantageous aspects, such as associated costs, ethics and time consumption [6]. For this reason, the use of rodents is a common choice in these trials even though many of their physiological and pathological processes greatly differ from the ones in humans.

In the last decades, the diurnal rodent *Octodon degus* (*O. degus*) has been claimed as an attractive tool for biomedical research in the field of age-related disorders, especially for neuroscience, since this species spontaneously develops protein aggregates in the brain [12,13,14]. Moreover, its social-affective behavior is of higher complexity than the one of mice or rats, and it shows as well other human-like disorders associated with the aging process, such as diabetes, retinal degeneration, tumors or endocrinal dysfunction [15,16]. Therefore, taking into account that aging is the main risk factor to develop sporadic PD (which represents the majority of cases) and that this disease co-occurs with other age-related alterations that influence its progression, if we were able to induce Parkinsonism in the *O. degus*, it could represent a promising scenario to investigate the physiopathology of the disease and how comorbidity influences its progression.

Taking this background as a starting point, the aim of this study was to investigate if the *O. degus* is sensitive to chronic MPTP-induced neurotoxicity and, thus, could be a suitable model for experimental Parkinsonism.

## 2. Results

### 2.1. MPTP Intoxication Affects Body Weight

**Figure 1:**
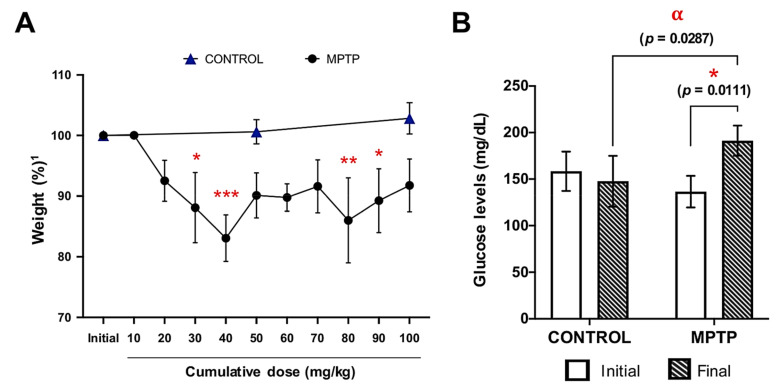
Weight and glucose levels are affected by MPTP intoxication. (**A**) Animals’ weight evolution along the experiment, expressed as percentage relative to their own initial weight^1^**.** Symbols: * at 30 mg/kg means *p* = 0.0122; *** at 40 mg/kg means *p* = 0.0001; ** at 80 mg/kg means *p* = 0.0018; * at 90 mg/kg means *p* = 0.0327). (**B**) Glucose levels were measured at two time points in both groups: before the MPTP injections started (initial) and the day the animals were euthanized (final). * Corresponds to *p* = 0.01 when initial MPTP vs. final day MPTP; α corresponds to *p* = 0.0287 when control final day vs. final day MPTP. Data are expressed as mean ± SD.

During the entire experiment, the weight of the animals was monitored (Figure 1A). In general, weight loss was observed as the dose of MPTP increased, and this decrease was very significant from the first (10 mg/kg) to the fourth dose (40 mg/kg). At this point, a little non-significant weight regain was observed, but a significant decrease was detected again at the time-point corresponding to doses of 80 and 90 mg/kg. No significant changes were observed in the weight of control animals (Figure 1A).

### 2.2. Increase in Glucose Levels Induced by MPTP Administration

Glucose levels of the two experimental groups were measured at the beginning of the experiment (one day before the injections started) and at the end of the experiment (Figure 1B). At baseline, there were no significant differences between the glucose levels of the control and MPTP groups. However, after MPTP intoxication, animals showed significantly higher glucose levels compared with both their baseline levels and with the control animals (Figure 1B).

### 2.3. MPTP Affects Motor Condition in the O. degus

**Figure 2:**
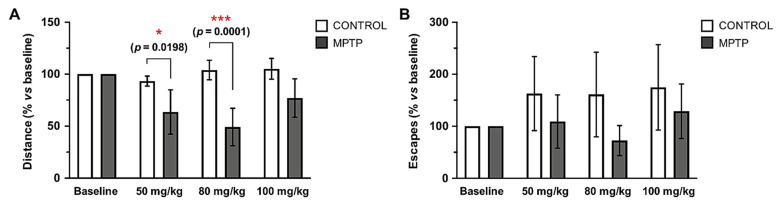
Traveled distance and escapes in the open field test decrease in the *O. degus* intoxicated with MPTP. Motor condition was evaluated by the actimeter test at four time points, taking the cumulative dose of MPTP as reference: before the MPTP injections started (baseline), 50 mg/kg, 80 mg/kg and 100 mg/kg. (**A**) Distance travelled by the animals expressed as a percentage in relation to its own baseline data. (**B**) Number of escapes from the arena of the open-field expressed as a percentage in relation to its own baseline data. Data are expressed as mean ± SD; * *p* < 0.05; *** *p* < 0.001.

The motor condition of the animals was evaluated at different time-points taking the accumulated doses of MPTP as time reference: baseline (prior to MPTP injections), and the doses of 50, 80 and 100 mg/kg. The traveled distance (cm) and the number of occasions they tried to escape from the platform were measured, and both parameters are expressed in % with respect to the baseline measurements of each group. Animals intoxicated with MPTP showed a decrease in the distance traveled as cumulative dose of MPTP increased, while control animals did not (Figure 2B). These differences between MPTP-intoxicated animals and control ones were significant and very significant at the doses 50 and 80 mg/kg, respectively, while at the dose 100 mg/kg a decrease in the distance traveled was still observed, but it did not reach statistical significance. Regarding the number of escapes, we observed that the control animals made more escapes within time, while the MPTP animals reduced the number of escapes as the MPTP dose increased, although no significant differences were found (Figure 2B). Surprisingly, in line with the data obtained in relation to spontaneous locomotor activity, at the 100 mg/kg dose, a recovery of motor capacity was observed.

### 2.4. O. degus Intoxicated with MPTP Show Cognitive Impairment

**Figure 3:**
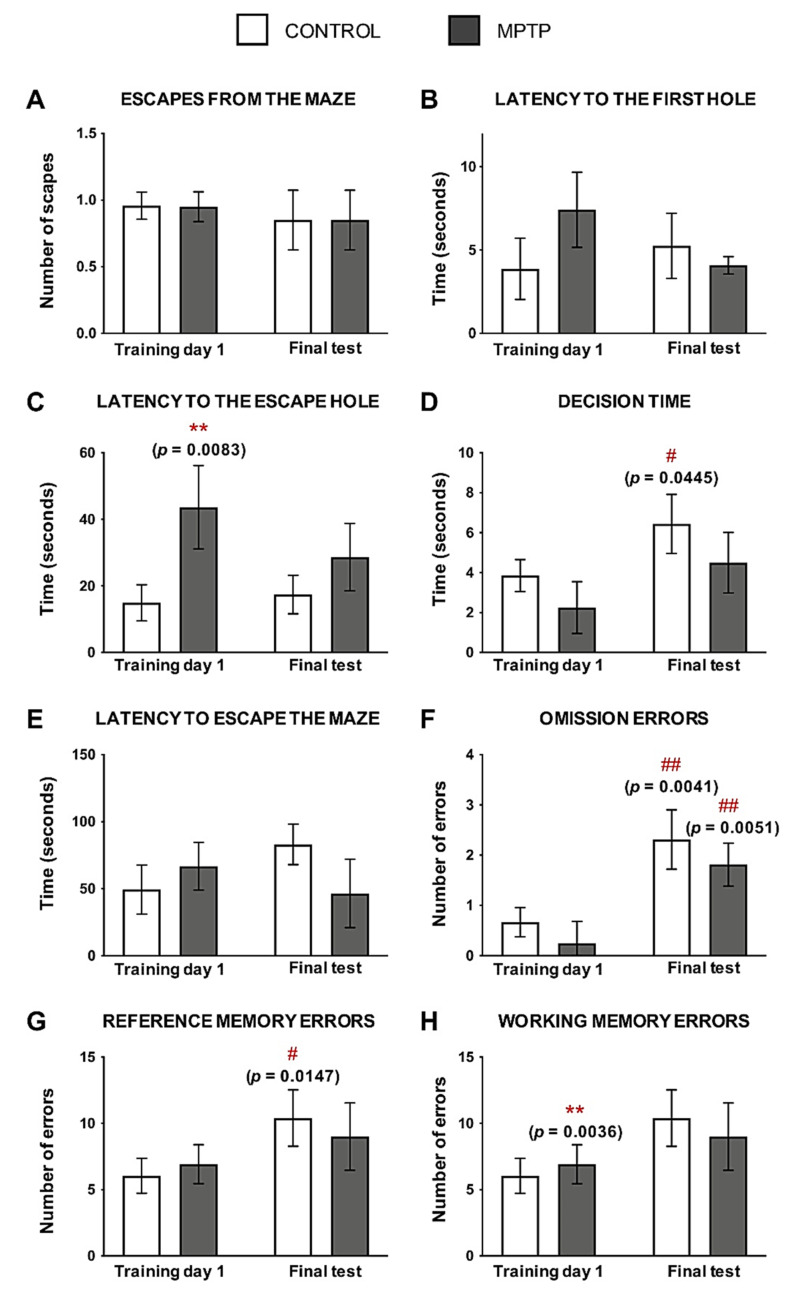
MPTP alters visuospatial memory in the *O. degus*. (**A**) Number of escapes from the maze. (**B**) Time spent (seconds) to find the first hole of the maze. (**C**) Time spent (seconds) to find the escape hole. (**D**) Time spent exploring the escape hole before leaving the maze. (**E**) Total time taken to escape the maze. (**F**) Number of visits to the escape hole without escaping through it. (**G**) Number of non-escape holes visited. (**H**) Number of repeated visits to non-escape holes. Data are expressed as mean ± SD; ** *p* < 0.01; Asterisks (*) indicate comparison between control and MPTP group in the same time point; pound sign (#) indicates comparison between the same group, different time point: control training day 1 vs. control final test and MPTP training day 1 vs. MPTP final test. # *p* < 0.05; ## *p* < 0.01.

In order to understand if MPTP intoxication had an effect on the cognition of these animals, the Barnes Maze test was performed when the animals had received a cumulative dose of 90 mg/kg. No significant differences were observed in relation to the number of times the animals escaped from the maze (Figure 3A), although the rest of the evaluated parameters showed differences when the two groups were compared (Figure 3B–H). 

Regarding the latency to find any hole, the MPTP animals reduced the time to find it after training, although no significant differences were found (Figure 3B). However, even if these animals improved this parameter with training, the time spent to find the escape hole was always higher when it was compared with the control group, reaching statistical significance in the first day of training (Figure 3C). 

The time spent exploring the escape hole was always shorter in the MPTP animals compared with the control group. We observed that, after training, animals from both groups reduced their decision time, and these differences were significant in the control animals when the two time points were compared (Figure 3D). 

The time spent to escape from the maze was higher in the control animals as the number of trainings increased, while the opposite occurred in the MPTP group (Figure 3E). 

Omission errors were significantly higher in the two groups of animals as they were trained, although these errors were lower in the MPTP group compared with the control one (Figure 3F). In order to evaluate the reference memory and the working memory, we evaluated the reference memory errors and the working memory errors, respectively. The same pattern was seen in reference memory errors (Figure 3G) and working memory errors (Figure 3H) than in the omission ones: errors increased with training, but the animals intoxicated with MPTP made fewer errors than the controls at the end of training.

### 2.5. Dopaminergic Alterations in the MPTP-Intoxicated Animals

**Figure 4:**
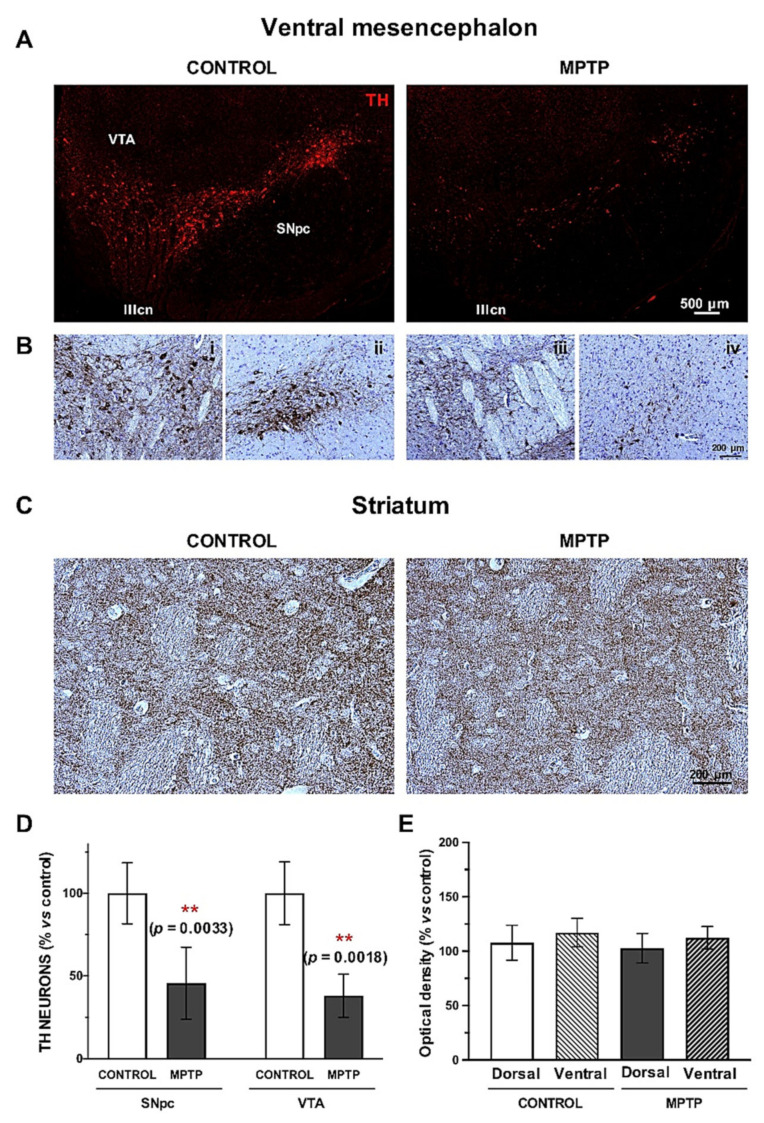
The dopaminergic pathway is compromised in the *O. degus* after chronic intoxication with MPTP. (**A**) Representative microphotographs of TH immunostaining performed in the ventral mesencephalon (SNpc and VTA) of control and MPTP animals. (**B**) TH immunohistochemistry counterstained with Nissl at the level of the exit of the third cranial nerve in VTA and lateral SNpc of control (i, ii) and MPTP animals (iii, iv). (**C**) Representative microphotographs TH immunostaining in the striatum of control and MPTP animals. (**D**) Quantification of TH+ neurons in the SNpc and in the VTA. (**E**) Optical density quantification of TH+ terminals by in the striatum. Data are expressed as mean ± SD; ** *p* < 0.01. SNpc = Substantia Nigra pars compacta; VTA = ventral tegmental area; IIIcn = exit of the third cranial nerve.

The dopaminergic system was studied by immunostaining of tyrosine hydroxylase (TH) in the ventral mesencephalon (SNpc and VTA), and in the striatum (Figure 4A,C).

We found a very significant decrease in the number of TH+ neurons both in the SNpc and in the VTA (Figure 4D), and this loss was slightly more accused in the VTA. Counterstaining with Nissl confirmed the dopaminergic cell death in the ventral mesencephalon (Figure 4B). In the striatum, we found a slight decrease in the optical density of the dopaminergic terminals in the MPTP group compared to the control, without significant statistical differences (Figure 4E). 

### 2.6. Increase of Neuroinflammatory Cells in the MPTP-Intoxicated O. degus

**Figure 5:**
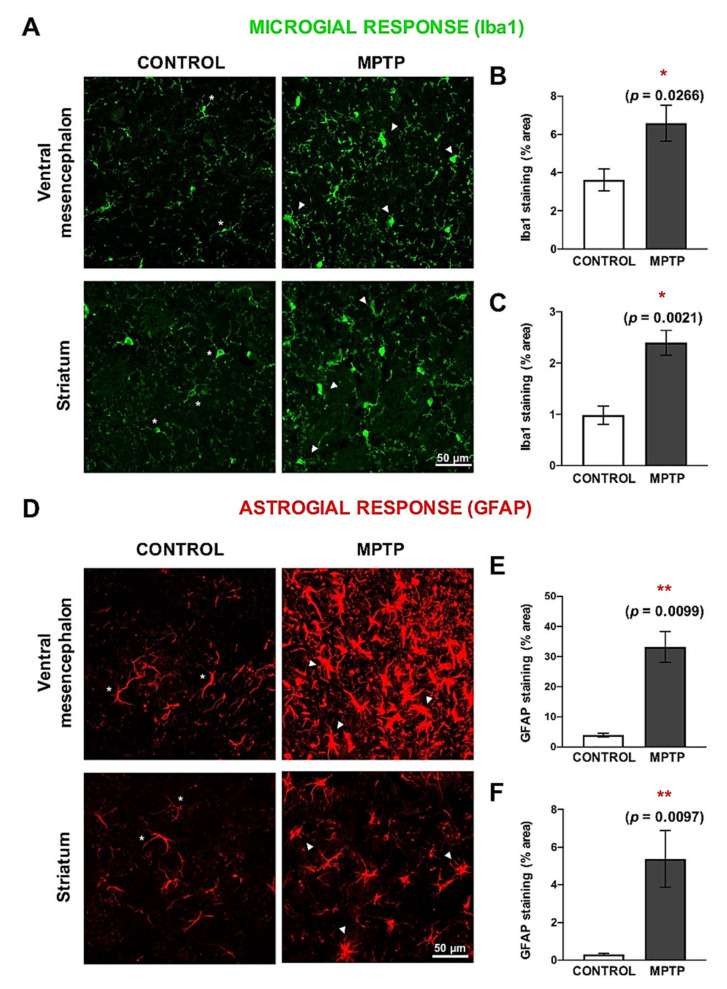
Neuroinflammation is exacerbated both in the ventral mesencephalon and in the striatum of MPTP-*Octodon degus*. (**A**) Representative microphotographs of microglial response, evaluated by Iba1 immunostaining. (**B**) Quantification of the Iba1^+^ surface (area expressed as % of the total area) in the ventral mesencephalon and (**C**) in the striatum. (**D**) Representative microphotographs of astroglial response, evaluated by GFAP immunostaining. (**E**) Quantification of the GFAP+ surface area (expressed as % of the total area) of the ventral mesencephalon and (**F**) in the striatum. White asterisks (*) indicate physiological-like morphology, while white triangles (△) indicate active or hypertrophic morphology. Data are expressed as mean ± SD; * *p* < 0.05; ** *p* < 0.01.

Possible MPTP-induced neuroinflammatory processes were studied by detecting microglia (Figure 5A–C) and astroglia (Figure 5D–F). In both, the ventral mesencephalon and the striatum, a very significant increase in the area occupied by these cells was observed (Figure 5B,C,E,F). Both microglial and astroglial responses were more exacerbated in the ventral mesencephalon than in the striatum. In addition, we observed clear morphological differences in the microglia and astrocytes when the two groups were compared. Regarding microglia, in the MPTP we found shorter processes and an ameboid-like shape (Figure 5A). Astrocytes found in in the control animals had thin GFAP+ branches, while in the MPTP one we detected hypertrophy and increase in the body size (Figure 5D).

### 2.7. MPTP Promotes Reactive Astrogliosis in the O. degus

**Figure 6:**
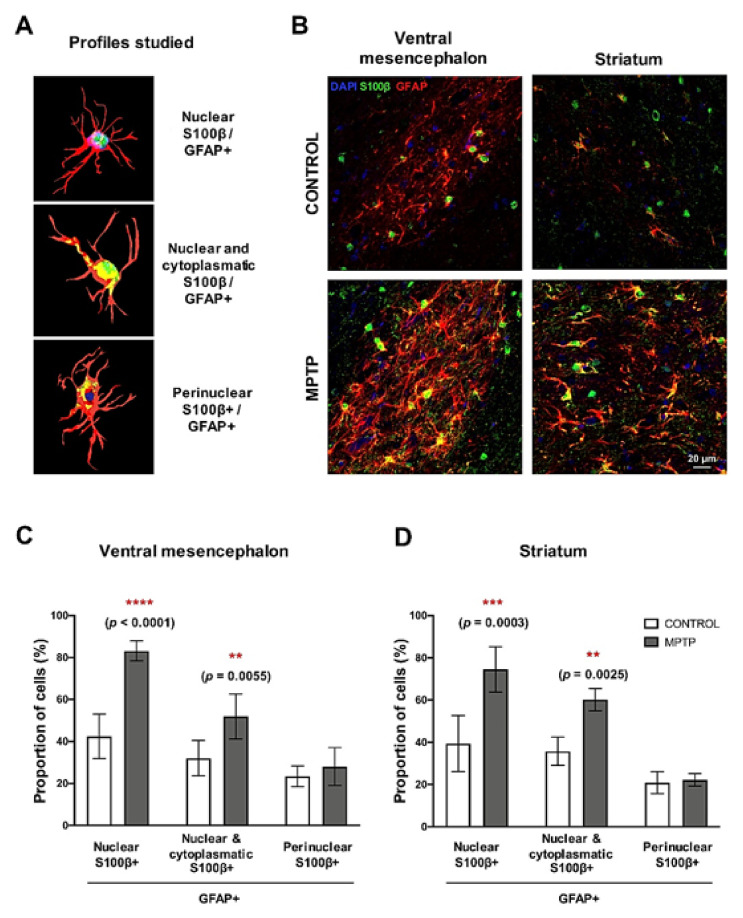
Changes in subcellular localization of S100β in astrocytes cells. (**A**) Schemes of the three profiles of GFAP+/S100β+ astrocytes studied. (**B**) Representative microphotographs of the GFAP/S100β immunostaining in the ventral mesencephalon and in the striatum. GFAP+/S100β+ astrocytes cell count in the (**C**) ventral mesencephalon and (**D**) in the striatum. Data are expressed as mean ± SD; ** *p* < 0.01; *** *p* < 0.001; **** *p* < 0.0001.

The calcium-binding protein S100β is mainly expressed by astrocytes, and it has been related to neuronal damage. For this reason, we studied different subpopulations of astrocytes according to the subcellular localization of S100β [17]. We identified three main types of GFAP+/ S100β+ cells (Figure 6A): (i) S100β exclusively co-localizing with DAPI (nucleus), (ii) S100β present in the nucleus and in the astrocyte’s cytoplasm and (iii) S100β outside the nucleus (perinuclear). We found that the together with the increase in GFAP expression, the pattern of distribution of S100β in the MPTP animals was different than the one in the control group (Figure 6B). In particular, we observed a very significant increase in the number of astrocytes with profiles ii and iii in both the ventral mesencephalon and in the striatum of the *O. degus* intoxicated with MPTP compared with the control group (Figure 6C). Interestingly, the increase in astrocytes with nuclear S100β was more significant than the rest of astrocytic profiles. We did not find significant differences in the number of GFAP+ cells with perinuclear S100β.

### 2.8. Increase of Microglial Cells in the Hippocampus

**Figure 7:**
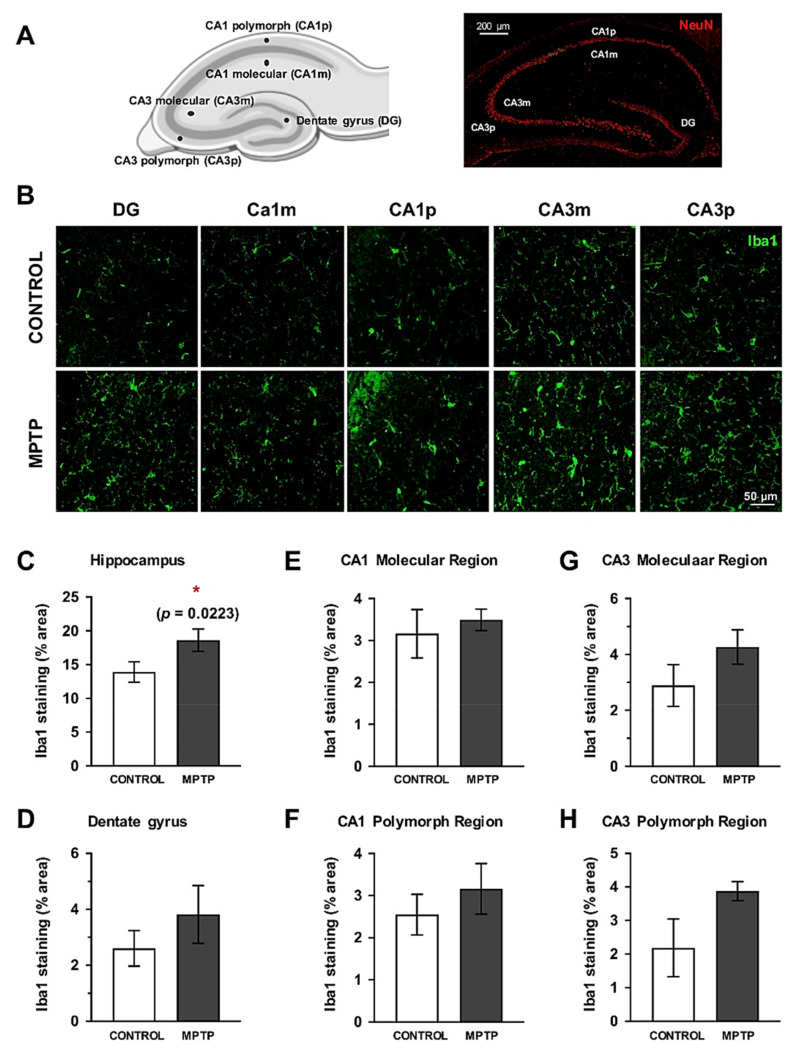
Microglial response is also increased in the dorsal hippocampus. (**A**) Schemes of the hippocampal areas that were considered for the analysis are indicated (created with https://biorender.com). (**B**) Representative images of the Iba1 immunostained sections. Quantification of the surface immunostained for Iba1 (area expressed as % of the total area) in the complete hippocampus (**C**), the dentate gyrus (**D**), CA1 molecular (**E**) and polymorph regions (**F**), CA3 molecular (**G**) and polymorph regions (**H**). Data are expressed as mean ± SD; * *p* < 0.05. DG = dentate gyrus, CA1m = CA1 molecular region, CA1p = CA1 polymorph region, CA3m = CA3 molecular region, CA3p = CA3 polymorph region.

Since cognitive alterations were found in the Barnes Maze test, we decided to analyze the inflammatory state in the dorsal hippocampus. For this, we studied the surface immunolabeled for Iba1 in the whole dorsal hippocampus and in its subareas: dentate gyrus, CA1 and CA3 (Figure 7A,B). When the analysis was performed on the entire hippocampus, a significant increase in the area occupied by Iba1+ cells was observed (Figure 7C). This increase was also detected in the individual area analysis, although the differences were not statistically significant (Figure 7C–H). Notably, the polymorphous areas had a greater increase than the molecular ones (Figure 7E–H).

## 3. Discussion

It has been more than 200 years since PD was first described, but given the complexity of the neurodegenerative process, many questions remain unsolved. Although there are many available therapies that improve the patients’ quality of life, they are not completely effective. In this sense, basic research and the use of experimental models are keys to understand the mechanisms that participate both in the onset and in the progression of the disease and, therefore, to develop diagnostic tools and accurate treatments.

It is widely accepted that a perfect experimental model has not been found yet; therefore, it is important to be aware of the advantages and limitations of the chosen experimental model. In this line, animal models for PD research are classically divided into genetic and toxin-based models [18]. Genetically engineered animals have provided valuable information on the role of specific proteins and metabolic pathways, especially for genetic forms of Parkinson’s disease. Their main disadvantage is that they are not able to induce dopaminergic nigral loss. On the other hand, models based on toxins often produce neuronal loss and decrease in dopamine levels, but they usually do not show the formation of Lewy bodies. Hence, a more ideal experimental scenario might be obtained by the combination of the two types of models, always keeping in mind that there is not a perfect experimental model of PD, but we can be able to use the most appropriate model that can answer a specific scientific question. 

**Figure 8:**
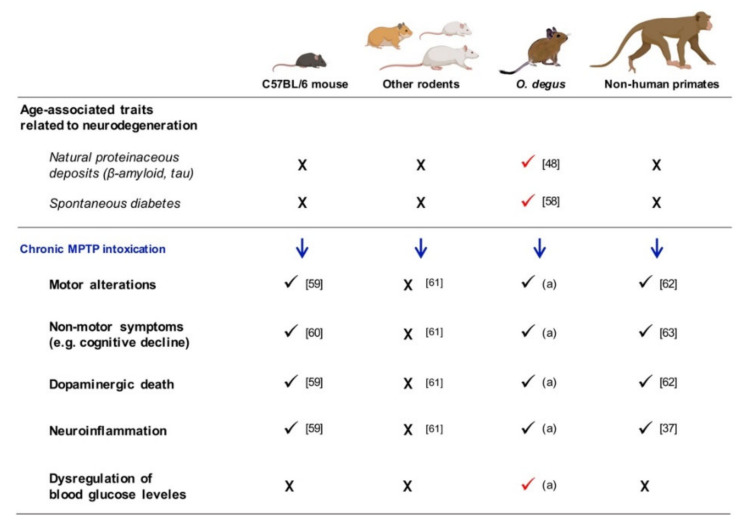
Summary of the effects of the chronic MPTP intoxication in different experimental models. “X” symbol indicates that the feature has not been found in the experimental model, while the check symbol means the presence of the feature. [19] Inestrosa et al., 2005; [20] Spear and Caple, 1984; [21] Luchtman et al., 2009; [22] Deguil et al., 2010; [23] Riachi et al., 1989. (a) Demonstrated in the present study; [24] Pérez-Otaño et al., 1994; [25] Vezoli et al., 2004.; [26] Barcia et al., 2004.

The experimental approach for PD based on the intoxication with MPTP is one of the most preferred because its use has provided important contributions to the understanding of idiopathic PD [18]. Since its discovery, the effects of MPTP have been widely described, and this prodrug is known to cause motor impairment, selective death of the dopaminergic neurons in the SNpc and loss of their striatal terminals, neuroinflammation and oxidative stress, both in humans and in experimental models (mainly non-human primates and mice) (Figure 8) [18]. Moreover, the effect of this neurotoxin can be modulated by different intoxication schedules and doses, offering the possibility to induce a variety of PD-like stages [6]. Among them, the chronic regimen (MPTP administration during several weeks) is the one that best reproduces the PD scenario regarding neuronal loss [18]. 

In this study, we have validated for the first time that the diurnal rodent *Octodon degus* is sensitive to the chronic intoxication with MPTP, by the examination of both behavioral performance and postmortem analysis.

In relation to the in vivo tests, we observed a significant reduction in the spontaneous exploratory movement of the animals within the cumulative dose of MPTP, although a slight increase in the locomotor activity was detected at the end of the experiment. This motor alteration directly correlates with the postmortem analysis of the dopaminergic system performed in the nigrostriatal pathway. The number of TH+ neurons in the ventral mesencephalon of MPTP-intoxicated *O. degus* was dramatically reduced: ~45% of survival in the SNpc and ~38% of survival in the VTA. Surprisingly, optical density of the dopaminergic striatal terminals remained unaffected. These results might explain the slight recovery observed in the motor performance of the MPTP animals in the last injection compared to the lower doses. In this line, previous studies have shown a spontaneous partial motor recovery after MPTP intoxication, which at the striatal levels corresponds with compensatory mechanisms in order to maintain the dopaminergic circuits [27,28,29]. This neuroadaptive changes can be dopamine-mediated, such as the promotion of dopamine re-uptake and promotion of striatal sprouting of the survival nigral neurons [30,31,32], but they can also be independent, such as the participation of external structures, serotonergic compensation or changes in the neuronal arborization in order to increase the postsynaptic contacts with the surviving neurons [27,33,34]. These findings are compatible with our findings. Despite that we performed several weekly injections, the last dose was time-spaced 8 days with the previous one. Therefore, during that time, phenomena of recovery could had taken place, and the last dose might have not been as strong as to provoke a significant decrease in the dopaminergic terminals. In this sense, future studies might focus on the characterization of the changes induced by MPTP intoxication, conducting different intoxication regimens and different time points for collecting the samples. Moreover, this model could be used to explore the compensatory mechanisms underlying the dopaminergic cell loss in PD.

On the other hand, the results concerning dopaminergic cell death in the mesencephalon were impressive to us since several lines of evidence have demonstrated that the TH+ VTA neurons are more resistant to the toxic effect of MPTP, and they generally appear unaffected [35]. However, in the MPTP-intoxicated *O. degus*, the dopaminergic neurons of the VTA were dramatically reduced, such as they were the SNpc ones. It is clear that more studies are needed to understand what are the differential elements in the response against MPTP intoxication in this species compared to other experimental models. An interesting line to explore could be the neuromelanin, calbindin or UCP2 content of these neurons [36,37,38,39]. Other emerging questions are the ones related to the catecholaminergic system: are other dopamine-innervated regions altered? Is the noradrenergic system affected in the cerebellar pathways [40,41]?

The increase in neuroinflammatory processes is a key hallmark of both PD and experimental models of the disease [10,42,43]. We also confirmed this feature in the MPTP-intoxicated *O. degus*, since a very significant increase was detected in the surface occupied by microglia (immunodetected with Iba1) and astroglia (immunodetected with GFAP), both in the ventral mesencephalon and in the striatum. This increment was more exacerbated at the ventral mesencephalon level. This behavior was also described by our group in MPTP-intoxicated monkeys 1 year after the injections had finished: the glial response was still significantly detected in the ventral mesencephalon but not in the striatum [26]. Moreover, this increase in the number of glial cells was accompanied by morphological changes in the degus administered with MPTP (reactive microglia and astroglia), according to what has been described in PD patients and experimental models of the disease [44]. To deepen in the inflammatory response, we studied the astroglial population regarding the subcellular distribution of S100β protein, which has been demonstrated to have an important role in PD [45,46]. We found that the number of astrocytes (GFAP+ cells) expressing S100β in the nucleus and in the cytoplasm was very significantly increased in the ventral mesencephalon and in the striatum of MPTP animals, as previously described in mice [17]. The increase in the number of GFAP+ cells with S100β+ cytoplasm supports its role as a regulator of proliferation and migration [47]. Our study was limited to explore the intracellular functions of S100β; however, it would be relevant to analyze extracellular concentrations of this protein because they have been described to be determinant to exert neuroprotection or detrimental effects [45]. 

Non-motor symptoms are also present in PD patients. In order to evaluate memory loss and cognitive performance, animals were subjected to the Barnes Maze test. The results obtained suggest visuospatial alterations in the performance of the intoxicated animals. MPTP animals had reduced the number of escapes from the maze compared with the control group, and they spent more time to find the escape hole. These findings might reflect the altered dopaminergic innervation in the VTA. However, it is interesting to see that after training, MPTP animals reduced the latency to find the first hole and also the escape hole, although their performance was still worse than the control animals one. This fact is important to explore new disease-modifying strategies in order to improve non-motor signs.

Control animals presented more errors (omission, reference and working memory errors) and spent more time to find the escape from the maze. These differences that were observed by comparison of the parameters of control and MPTP animals might reflect alterations in the dopaminergic system: after training, control animals spent more time exploring the maze and deciding whether to exit or not. On the contrary, MPTP animals seemed more impulsive (related to impaired dopaminergic circuits) and made fewer errors because they did not explore the maze. These behaviors could also be due to a different motivation to escape, to motor alterations or to the development of different strategies to solve the maze. This result can be explained by the fact that animals were not as afraid as in the beginning, as previously explained by our group [48]. However, at the end, control animals were able to find the escape hole before the MPTP ones because they were able to remember it, and they made more correct answers. These findings represent a novelty because the previous published works have only detected cognitive alterations at low MPTP doses.

Once we had detected these alterations in visuo-spatial memory and the loss of dopaminergic neurons in the VTA, we decided to study neuroinflammation in the hippocampus, as it is a key brain area for the memory processes [49]. Other studies have demonstrated that, after MPTP intoxication, the hippocampal neuronal population was not altered, but microgliosis is detected [50,51,52]. Accordingly, we observed an increase in the area occupied by Iba1+cells (trend). Therefore, this scenario could be interesting to study, including the role of the hippocampus on PD and how it influences the progression of the neurodegenerative process, although a deeper characterization of the nature of the inflammatory response should be carried out. 

Finally, we also studied blood glucose levels because increasing evidence is highlighting the link between diabetes and PD [53]. We found that MPTP produced an increase in systemic glucose levels in the intoxicated degus, compared with the control animals. In other words, MPTP seems to dysregulate glucose levels, either through dopaminergic denervation or by another mechanism. This finding adds a pathological characteristic to this model that can make a high translational impact. The *O. degus* naturally develops type II diabetes, and since glucose levels were more dysregulated after MPTP intoxication, this species offers the possibility to study the co-occurrence of type II diabetes and induced experimental Parkinsonism.

Different authors have shown that these animals naturally accumulate neuropathological proteins, such as β-amyloid deposits and neurofibrillary tangles of phosphorylated tau protein, as well as other signs of neurodegeneration [19,54]. In terms of PD pathology, only one study has been carried out to find characteristic hallmarks, but the authors failed to detect α-synuclein deposits or loss of neuronal density in old specimens of *O. degus* [55]. However, this discrepancy might be due to the influence of external factors that could favor neuropathological hallmarks, or even the methodology used was not able to detect non-fibrillary aggregates [14]. In addition, these animals were not induced to Parkinsonism. Within this context, could MPTP accelerate these protein deposits in the *O. degus’* brains or even provoke the formation of Lewy body-like structures? Future investigations (probably including aged animals) could orientate to better characterize this model.

The relevance of these results is also supported by the fact that the *O. degus*, differently to rats and mice, is a diurnal rodent that has been classically considered as a natural model to study neurodegeneration because it spontaneously develops cognitive decline, Alzheimer’s disease-like histopathological hallmarks, retinal and macular degeneration [14].

Additionally, this animal shows some age-related traits such as spontaneous tumors, kidneys dysfunction or electrocardiographic alterations (unpublished data of our lab) [56,57]. Therefore, the use of this model offers the possibility to set a more realistic scenario related to the complex process of aging, and also to test therapeutic strategies that could slow down the neurodegenerative process [49].

Finally, despite the fact that MPTP has been well characterized to generate animal models that mimic human PD, most of the rodents show low susceptibility to MPTP, or even complete resistance, such as golden hamsters, rats and some mouse strains (e.g., SWR/J and AKR/J) (Figure 8) [58,59,60]. In contrast, we have demonstrated that the rodent *O. degus* is sensitive to the neurotoxic effect exerted by MPTP. Then, some questions arise: is it due to the integrity of their blood brain barrier and the cerebrocapillary endothelium? Do they have lower content of MAO-B in the peripheral tissues for MPTP systemic clearance? Is their glymphatic system altered (as it happens in human patients of AD), and for this do they also accumulate pathological proteins? Does the extracellular matrix play a role in this response in the *O. degus*? [61,62]. Numerous lines of research can be developed to solve all these questions and hopefully would uncover the human susceptibility to the development of sporadic PD.

### Limitations of the Study

Our experimental design allowed us to confirm that the *O. degus* is sensitive to MPTP intoxication, but we encountered some limitations. In the first place, although some results are consistent enough, others suggest that increasing the number of animals would have helped us to reduce heterogeneity among individuals. For example, the increase in the inflammatory response in the hippocampus was not found statistically significant in the hippocampal subareas, although analyzing the complete hippocampus showed a significant increase in neuroinflammation. Another point we would like to emphasize is that it would be interesting to perform experiments of molecular biology, such as the determination of MPP+ levels in the striatum or the study of the suggested striatal nerve recovery. We hope that the present results will inspire future lines of research and these questions would be addressed.

## 4. Materials and Methods

### 4.1. Animals 

Ten juvenile male *O. degus* (1 year old, 225–280 g at the beginning of the experiment) were randomly divided into two experimental groups before motor state and behavior were assessed: i) control group (*n* = 5) and ii) MPTP group (*n* = 5). The *O. degus* belonged to Prof. Herrero’s colony in the animal house of the University of Murcia-CEIB. They were housed in groups of 2–3 individuals inside transparent cages that were located in a room with controlled temperature (22 ± 1 °C), humidity (60%), and 12 h light-dark cycle (lights were automatically turned on at 08:00 a.m.) with free access to water and food (Harlan Teklad Global Diet^®^, Harlan Laboratories, Indianapolis, IN, USA). All experimental procedures using animals were carried out following the European Community Council Directive (2010/63/UE) and were approved by the ethical committee of the University of Murcia (project number: A13170102 / CEEA-OH AMP/MOD 103/2014 + 2018).

### 4.2. MPTP Administration and Quarantine Period

*O. degus* from the MPTP group received two MPTP injections per week (10 mg/kg/injection, i.p., Sigma-Aldrich-Merck, St. Louis, MO, USA). This procedure was repeated for five weeks, until the animals reached a cumulative dose of 100 mg/kg. Injections were performed with 3–4 day intervals, except for the last injection, which we left 8 days from the 9th to the 10th injection in order to make compatible the performance of the Barnes Maze test with the day that the animals were euthanized. This procedure was adopted because we were interested in the study of neuroinflammation, and it has been described that in other experimental models this process reaches the highest point 48 h after the last MPTP injection [10]. 

For the injections, animals were anesthetized intramuscularly with a cocktail of ketamine (75 mg/Kg, Anesketin^®^ 100 mg/mL, Dechra Veterinary Products SLU, Barcelona, Spain) and medetomidine (0.5 mg/Kg, Domtor^®^1 mg/mL, Ecuphar^®^, Barcelona, Spain). The responsible investigator followed the safety protocols described by Jackson-Lewis and Przendborski [63]. In order to ensure that all the animals received 10 mg/kg in every injection, doses were recalculated before each MPTP injection by weighting *O. degus*. The measurement of the weight before each injection allowed us to evaluate how the previous one had affected the weight of each animal. After MPTP intoxication, animals were placed inside cages with a negative pressure system (IVC transport rack, Techniplast, Buguggiate, Italy), and 48 h later (security period) they were returned to their original cages. Additional animal care was considered during the security period: heat lamps, hydration gel (gel diet water Safe^®^) and food placed in the floor of the cage to make it more accessible. Knowing that MPTP neurotoxicity is not exacerbated by the use of anesthetics, control animals were not anesthetized in order to avoid additional invasive procedures.

### 4.3. Behavioral Tests

For the evaluation of motor and cognitive conditions, animals were subjected to two well-characterized procedures in rodents: the open field test and the Barnes Maze. All procedures were performed between 9 a.m. and 2 p.m. in a quiet room.

1. Open field test. The open field test is one of the most common locomotor tests for experimental PD because rodents with motor deficits or cognitive decline can show an impaired ability to move and explore the arena [64]. The chamber consisted of a transparent open Plexiglas arena (44.5 cm wide × 44.5 cm long × 40 cm high) located inside a system of two sets of 16 infrared photocells (SAI Electronics & Engineering division, University of Murcia, Murcia, Spain). Horizontal locomotor activity and vertical movements were measured by the registration of the beam breaks, which were recorded and transmitted to a computerized system [65]. Every animal was placed in the center of the arena, and its activity was recorded during 180 s. The chamber was cleaned with 70% ethanol after each trial. The test was done taking the following MPTP injections as a time reference: baseline (prior to the first MPTP injection), 48 h after the fifth MPTP injection (cumulative dose of 50 mg/kg), the eighth MPTP injection (cumulative dose of 80 mg/kg) and the last MPTP injection (cumulative dose of 100 mg/kg). Control animals performed the test the same days in order to have comparable measures.
2. Barnes Maze. This cognitive test is performed in a Plexiglass circular platform (160 cm diameter) that contains 18 holes: 17 of them are blocked with a mesh, and one is the exit of the maze to the animal’s cage. The test consists of three phases: (i) the habituation day (day 0), in which the animal is kindly taught by the investigator which is the path to escape the maze from the center of the platform; (ii) the training days (days 1–7), in which the animal is placed in the center of the platform and has 4 min to escape the maze (this is repeated for 4 rounds); and (iii) the retention day (“final test”, day 8) that follows the same protocol as the training days. During the training and the retention days, we measured several parameters to evaluate spatial memory. For a detailed description of the protocol, see [66]. Barnes Maze was performed before the last MPTP injection.

### 4.4. Glucose Measurement

Glucose levels in the blood were determined in vivo with a glucometer (Accu-Chek Compact Plus, Roche, Basel, Switzerland) one week before the MPTP injections started and just before the animals were euthanized. For this procedure, *O. degus* were anesthetized with isoflurane, and blood was taken from the femoral vein by puncture. 

### 4.5. Samples Obtention and Brain Tissue Preparation

Forty-eight hours after the last MPTP injection, animals were euthanized. First, degus were anesthetized with isoflurane for blood collection and glucose level measurement. Then, animals were introduced into a CO_2_ flux chamber, and brains were removed and fixed in 4% paraformaldehyde in 0.1 M phosphate buffer saline (4% PFA in PBS, pH = 7.4) for 48 h, RT. After, brains were washed in distilled water (10 min, 3 times) and immersed in absolute ethanol until they were embedded in paraffin blocks.

Postmortem analyses were performed in 5 µm coronal brain sections, obtained with a microtome (Thermo Scientific HM 325 Rotary Microtome, Thermo Fisher Scientific, Waltham, MA, USA). The studies were performed in the striatum (at the anterior white commissure level), in the ventral mesencephalon (Substantia Nigra pars compacta, SNpc, and the Ventral Tegmental Area, VTA) and in the dorsal hippocampus. Brain areas were identified according to the mouse brain atlas [63]. 

### 4.6. Immunohistochemistry and Immunofluorescence

The sections were dewaxed in the oven (2 h, 65 °C) and by immersion in xylene (10 min, RT). Then, they were hydrated by immersion in decreasing concentrations of ethanol (100, 95 and 80%) and finally in distilled water. The antigen retrieval was performed using citrate buffer solution (pH = 6.0, 20 min, 95 °C), and the sections were washed in PBS (2 × 3 min, RT). 

In the samples for immunohistochemistry, endogenous peroxidase was blocked with 0.3% hydrogen peroxide (20 min, RT), and unspecific bindings were blocked with 10% goat serum (in PBS + Triton 0.5%, 30 min, RT, S-1000 Vector Laboratories, Burlingame, CA, USA). Primary antibody was applied overnight at 4 °C: mouse monoclonal TH (1:500 in PBS + Triton 0.5% + serum 1%; MAB318 Sigma-Aldrich, St. Louis, MO, USA). Sections were washed with PBS and incubated with a biotinylated anti-mouse secondary antibody (1:250, BA-9200-1.5, Vector Laboratories, 1h, RT). Then, a signal amplifier was applied following the manufacturer’s indications (ABC Elite Kit, Vectastain, Vector Laboratories). Sections were incubated with DAB substrate until reaction occurred (DAB Peroxidase HRP Substrate Kit, Vector Laboratories); finally, then they were dehydrated and mounted with cover-slip and DPX (Sigma-Aldrich). For TH immunohistochemistry in the ventral mesencephalon, sections were counterstained with Nissl.

The samples for immunofluorescence staining were blocked with 10% animal-free blocker (in PBS + Triton 0.5%; SP-5035 Vector Laboratories) and then incubated overnight at 4 °C with the corresponding primary antibodies diluted in PBS + Triton 0.5% + serum 1%: Iba1 (1:1000, ab178846 Abcam), GFAP (1:500, MAB360 Sigma-Aldrich), NeuN (1:500, MAB377 Sigma-Aldrich) and S100β (1:500, ab52642 Abcam). After PBS washes (3 × 5 min, RT) secondary antibody was placed in the sections following the manufacturer’s instructions (VectaFluor™ Duet Immunofluorescence Double Labeling Kit, Vector Labs). Then, the slices were washed in PBS (6 × 5 min, RT) and finally cover-slipped (VECTASHIELD Antifade Mounting Medium, Vector Laboratories).

### 4.7. Image Caption and Stereological Analysis

The samples from immunohistochemistry were photographed with the Hall 100 ZEISS microscope. Series of 8 images (100 µm apart each) of the mesencephalon stained for TH were taken at 10× magnification, covering the entire SNpc and the VTA (from rostral to caudal axis). Both hemispheres were photographed, and 4 replicates per area and sample were taken. Then, nuclei surrounded by TH+ immunostaining were counted by an automated plug-in of Fiji software in order to avoid bias in the cell count (based on the optical fractionator) [67]. Niss+ nuclei were also studied to ensure accurate neuronal count. 

The striatal dopaminergic terminals were determined in the striatum using 6 brain slices per animal. The striatum was divided into two areas: ventral and dorsal. In each slice, four images per hemisphere and striatal area were taken at 20× magnification. Quantification of the TH immunoreactivity in striatal fibers was assessed by optical density using Fiji software, applying a plug-in for color deconvolution for DAB stainings [9,67]. 

The study of inflammatory markers was performed by immunofluorescence in the ventral mesencephalon (at the level of the exit of the third cranial nerve, IIIcn), striatum (anterior white commissure level) and in the dorsal hippocampus (dentate gyrus, CA1 and CA3; polymorphous and granular regions). The images were taken with a confocal microscope using a 40× oil objective (microscope Leica TCS-SP8, SACE, University of Murcia, Murcia, Spain). We selected the upper and lower limits in the z-position mode to acquire a series of 10 images at 0.5 µm intervals. At the ventral mesencephalon and at the striatum levels, both hemispheres were photographed (3 images per each, 2 slices per animal). At the hippocampal level, two images per subarea were taken from both hemispheres of each animal (2 brain slices/animal). Quantification of Iba1 and GFAP immunolabeling was done by measuring the area occupied by each marker in the z-stack images and expressed relative to the total area (in percentage, %) [35]. The quantification of S100β+ astrocytes was done by counting the number of cells expressing three defined profiles: nuclear S100β, nuclear and cytoplasmic S100β and perinuclear S100β. 

Importantly, all the images taken for the same analysis were made and quantified in the same conditions (including light and room temperature, and without editing them for analysis) in order to avoid bias. 

### 4.8. Statistical Analysis

Normality of all the data was tested by the Kruskal–Wallis test. Then, the statistical analysis to compare groups and data sets was chosen depending on the number of variables studied, considering in all cases values *p* < 0.05 as significant. One-way ANOVA followed by the post hoc Tukey test was applied to the data regarding weight and optical density of the striatum. Data of glucose levels, open-field, Barnes Maze test and S100β immunofluorescence were analyzed with a two-way ANOVA followed by the post hoc Tuke testy. Data obtained from the immunohistochemical and immunofluorescence stainings (except from S100β) were analyzed with a *t*-test. 

## 5. Conclusions

After chronic MPTP intoxication in *O. degus*, we have detected the following features: dysregulation of blood glucose levels, both motor and cognitive alterations, dopaminergic neuronal death and increase in the inflammatory processes in the nigrostriatal pathway and in the hippocampus. These novel results suggest that the *O. degus* could represent a new natural tool in the research of age-associated PD, although future in-depth characterization is needed. Therefore, this study offers a new alternative to study PD from the perspective of aging and co-morbidities, closer to the human situation. 

## Data Availability

Upon request, the authors can provide the raw data obtained in the present study.

## Abbreviations

DG: dentate gyrus; MPTP, 1-methyl-4-phenyl-1,2,3,6-tetrahydro-pyridine; *O. degus*, *Octodon degus*; PD, Parkinson’s disease; SNpc, Substantia Nigra pars compacta; VTA; ventral tegmental area.
